# A panoramic driving perception fusion algorithm based on multi-task learning

**DOI:** 10.1371/journal.pone.0304691

**Published:** 2024-06-04

**Authors:** Weilin Wu, Chunquan Liu, Haoran Zheng

**Affiliations:** 1 Guangxi Applied Mathematics Center, College of Electronic Information, Guangxi Minzu University, Nanning, China; 2 Guangxi Postdoctoral Innovation Practice Base, Wuzhou University, Wuzhou, China; 3 Faculty of Engineering, Chemical and Materials Engineering, University of Auckland, Auckland, New Zealand; Anhui University, CANADA

## Abstract

With the rapid development of intelligent connected vehicles, there is an increasing demand for hardware facilities and onboard systems of driver assistance systems. Currently, most vehicles are constrained by the hardware resources of onboard systems, which mainly process single-task and single-sensor data. This poses a significant challenge in achieving complex panoramic driving perception technology. While the panoramic driving perception algorithm YOLOP has achieved outstanding performance in multi-task processing, it suffers from poor adaptability of feature map pooling operations and loss of details during downsampling. To address these issues, this paper proposes a panoramic driving perception fusion algorithm based on multi-task learning. The model training involves the introduction of different loss functions and a series of processing steps for lidar point cloud data. Subsequently, the perception information from lidar and vision sensors is fused to achieve synchronized processing of multi-task and multi-sensor data, thereby effectively improving the performance and reliability of the panoramic driving perception system. To evaluate the performance of the proposed algorithm in multi-task processing, the BDD100K dataset is used. The results demonstrate that, compared to the YOLOP model, the multi-task learning network performs better in lane detection, drivable area detection, and vehicle detection tasks. Specifically, the lane detection accuracy improves by 11.6%, the mean Intersection over Union (mIoU) for drivable area detection increases by 2.1%, and the mean Average Precision at 50% IoU (mAP50) for vehicle detection improves by 3.7%.

## Introduction

In recent years, under the guidance of the low-carbon economy concept, the global automotive industry is continuously developing towards energy diversification, intelligence, and greening. With the advent of the 5G era, the development of intelligent connected vehicles has been greatly promoted. At the same time, it also has higher requirements for the level of intelligence of existing vehicles. With the continuous evolution of intelligent connected vehicle technology, panoramic driving perception systems are also constantly advancing as one of its key components The panoramic driving perception system senses the surrounding environment of the vehicle through a variety of sensors (such as cameras, lidar), providing reliable data support for intelligent connected vehicles. However, in practical applications, considerations extend beyond accuracy and robustness to computational efficiency and overall performance in order to meet the application requirements of low-cost autonomous driving. Within the panoramic driving perception systems framework, lane line detection, drivable area detection, and vehicle detection represent pivotal technological tasks.

In the realm of lane detection, traditional computer vision algorithms are progressively being supplanted by deep learning methodologies. Conventional lane detection techniques typically hinge on computer vision technologies, such as edge detection and morphological transformations. In contrast, deep learning accomplishes more precise lane detection by extracting more intricate image features, as exemplified by LaneATT [1], Enet [2] (Efficient Net), and LATR [3] (LAne detection TRansformer), among others. As for drivable area detection, the use of multi-scale algorithms like Swin-APT [4] (Swin-Transformer Adaptor for Intelligent Transportation) and DeepLabv3+ [5] can effectively augment its accuracy and robustness. In the realm of vehicle detection, the advent of deep learning has seen a gradual replacement of traditional vehicle detection methods with deep learning networks, including EnsembleNet [6], You Only Look Once (YOLO), and Swin Transformer [7]. While these tasks individually exhibit excellent performance, their combined performance often needs to be improved, struggling to balance accuracy and robustness. The YOLO series of object detection algorithms, such as You Only Look Once version 5 [8] (YOLOv5) and You Only Look Once version 8 [9] (YOLOv8), as current mainstream detection algorithms, provide clear directions for the multi-task learning network in this paper.

In order to enhance the performance and practicality of panoramic driving perception systems, numerous studies have been dedicated to designing more efficient and accurate multi-task learning networks. In recent research, multi-task learning networks such as DeMT [10] (Deformable Mixer Transformer), SMNet [11] (Symmetric Multi-task Network), YOLOP [12], HybridNets [13], and YOLOPv2 [14] have gradually integrated single tasks into multi-tasks and processed them simultaneously to impove performance. However, these multi-task learning networks still face certain challenges in current low-cost autonomous driving applications. The design of a high-performance, small-parametric multi-task learning network that is suitable for traffic scenarios is an urgent problem that requires immediate attention.

Most modern cars are equipped with single-type sensors, providing crucial data sources for automotive driver assistance systems. However, any system that relies on a single sensor as its only data source involves a trade-off between advantages and shortcomings. For example, Tesla relies solely on cameras for assisted driving, which leads to poor environmental adaptability. It is easily disturbed by weather, such as rain, snow, fog, and dust, and cannot meet the requirements for autonomous driving in different weather conditions and at higher speeds.

Therefore, a single sensor cannot resolve all issues, and data fusion from multiple sensors is inevitably a trend. Multi-sensor fusion mainly includes pixel-level fusion, feature-level fusion, and decision-level fusion [15]. Common fusion methods include wavelet transform methods [16], clustering methods [17], and logical theory [18]. By fusing data from multiple sensors, the accuracy of detection and the system’s safety can be effectively improved. This approach is less affected by the limitations of sensors, thereby enhancing the performance of the car’s driver assistance system.

Addressing the aforementioned issues, this paper proposes a panoramic driving perception fusion algorithm based on multi-task learning, which comprehensively handles multiple tasks such as lane detection, drivable area detection, and vehicle detection. It adopts a multi-sensor fusion strategy, specifically, the fusion of lidar and visual sensors, to achieve synchronization of different sensors in time and space, enhancing the accuracy and robustness of the panoramic driving perception system. Furthermore, it bolsters computational efficiency and overall system performance, bearing significant implications for low-cost autonomous driving applications.

The main contributions of this paper can be summarized as follows:

1. This paper proposes a panoramic driving perception fusion algorithm based on multi-task learning. This algorithm enables the simultaneous processing of multi-task and multi-sensor data. It achieves feature-level fusion of lidar and visual sensors, leading to a comprehensive enhancement in the driving perception performance of vehicles. Additionally, it presents a feasible technical solution for autonomous driving.
2. In order to address the limitations of the YOLOP network, this study introduces the C2f, SPPF, and ConvTranspose2D structures. These structures are aimed at improving the adaptability of the feature map pooling operation and minimizing the loss of details during downsampling. Through optimization of the original network structure and loss function, the paper effectively resolves the issues related to adaptability and detail, thus significantly enhancing the detection performance and robustness of the multi-task learning network.
3. A data fusion algorithm for lidar and visual sensors is devised to overcome the limitations of using a single sensor. This algorithm reduces the redundancy of sensor data, facilitates the sharing of perception information between sensors, and leads to a substantial improvement in the performance and accuracy of perception information subsequent to multi-sensor data fusion.

## Related content

### Multi-task learning network-YOLOP

**Fig 1:**
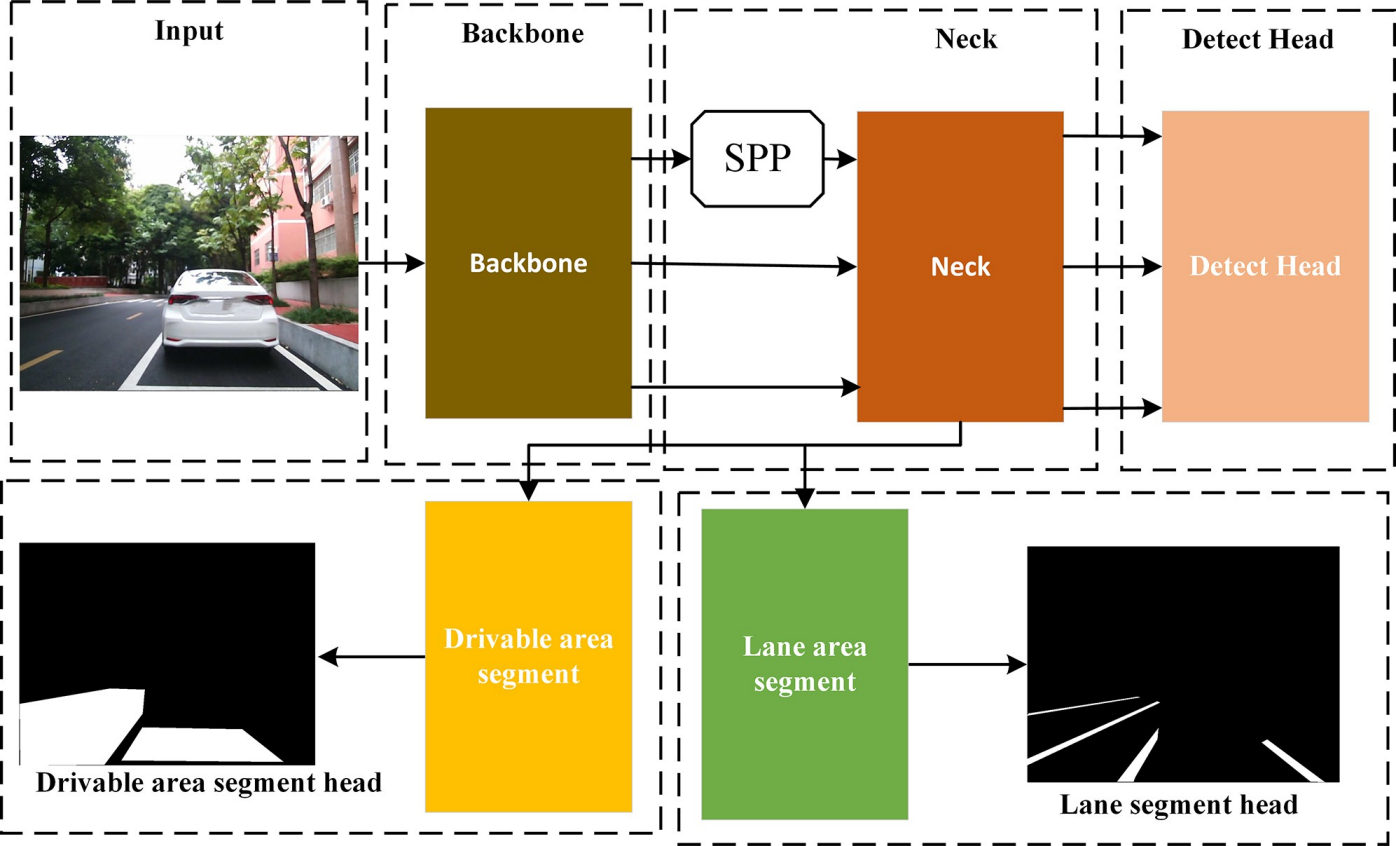
Network structure of YOLOP.

YOLOP network structure mainly consists of an encoder and three decoders. The encoder is used to extract target features, while the decoders are used for target detection. The network structure is shown in Fig 1.

The YOLOP network comprises two main parts: the encoder and the decoder. It utilizes the Cross Stage Partial Darknet [19] (CSPDarknet) as the Backbone network, while the Neck network comprises Spatial Pyramid Pooling [20] (SPP) and Feature Pyramid Network [21] (FPN). The decoder is composed of a vehicle detection head, a drivable area segmentation head, and a lane line detection head. They each receive the output features of the encoder for downsampling and upsampling, and through a series of convolutional layers and Bottleneck Cross Stage Partial [22] (BottleneckCSP) modules, the detection of drivable areas and lane lines is achieved. Finally, the prediction results are divided into multiple scales to improve detection accuracy.

The entire YOLOP network structure allows for end-to-end training, where it can directly input images and obtain the results of the three tasks in the output. Therefore, based on YOLOP, this paper addresses the issue of sub-optimal network performance by improving various modules and optimizing the network structure. This achieves higher accuracy and robustness, making it perform better in practical applications.

### Lidar and vision joint calibration

In processing raw lidar point cloud data, the inherent sparsity poses significant challenges. Utilizing time-synchronized image data effectively addresses this challenge. For precise object perception, the point cloud data should be projected onto the image, ensuring overlap. This operation mandates a joint calibration between lidar and vision sensors. Consequently, leveraging the detailed information from image data, combined with point cloud data accuracy, augments object detection and classification performance.

In many vehicular systems, the acquired lidar data predominantly comprises parameters such as horizontal rotation angle and distance. For enhanced fusion of lidar and vision data, conversion of point cloud data from polar coordinates (angle and distance) to Cartesian coordinates (x, y, z) is imperative.

**Fig 2:**
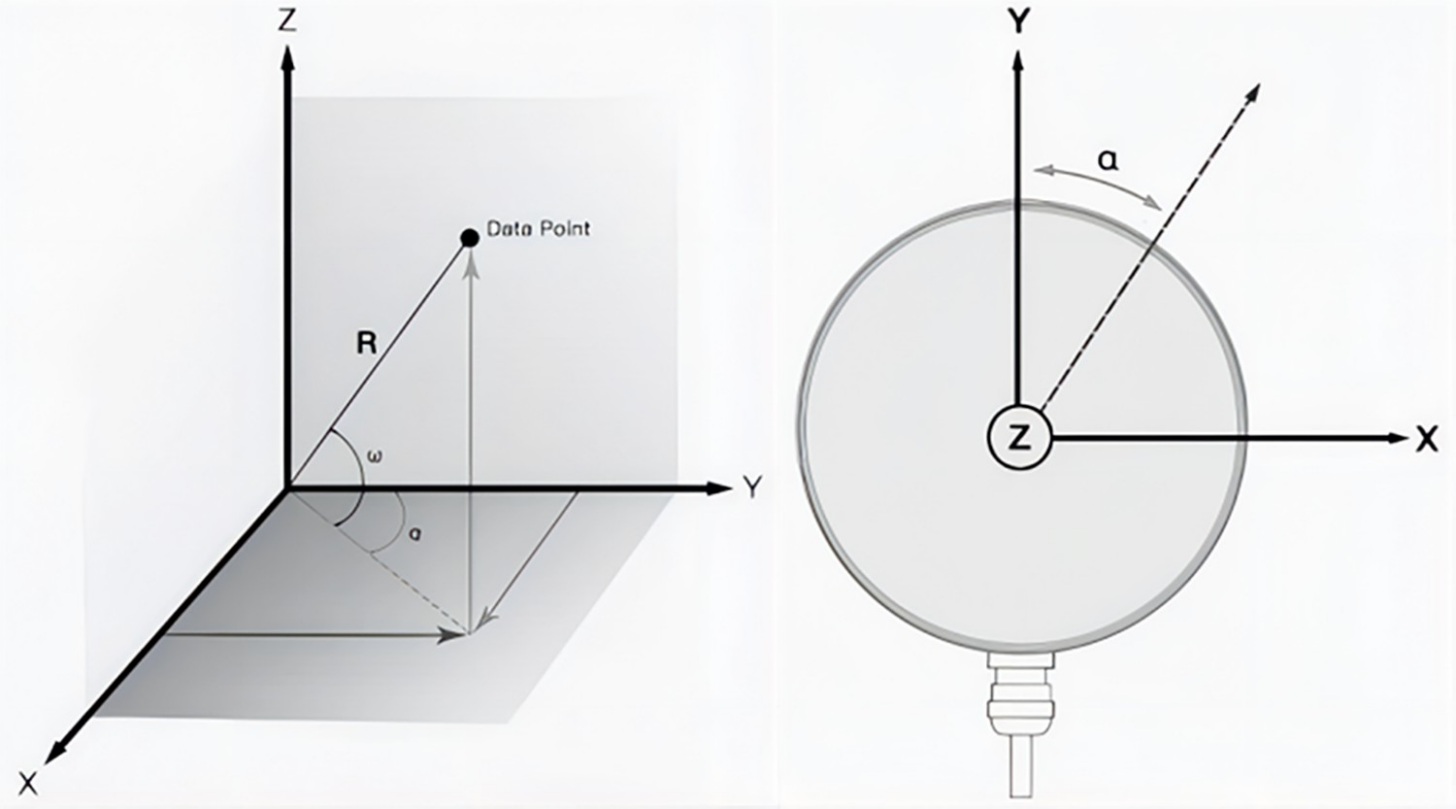
Mapping of lidar polar coordinates and XYZ coordinates.

The distribution of the coordinates is shown in Fig 2, and their conversion relationship can be expressed as:

$$\left\{ \begin{array}{l} x=r\;\cos(\omega )\sin(\alpha );\\ y=r\;\cos(\omega )\cos(\alpha );\\ z=r\;\sin(\omega ); \end{array}\right.$$ (1)

Among them, *r* is the measured distance, *ω* is the vertical angle of the lidar, and *α* is the horizontal rotation angle of the lidar. The coordinates *x*, *y*, *z* represent the projections of polar coordinates onto the X, Y, Z axes respectively.

**Fig 3:**
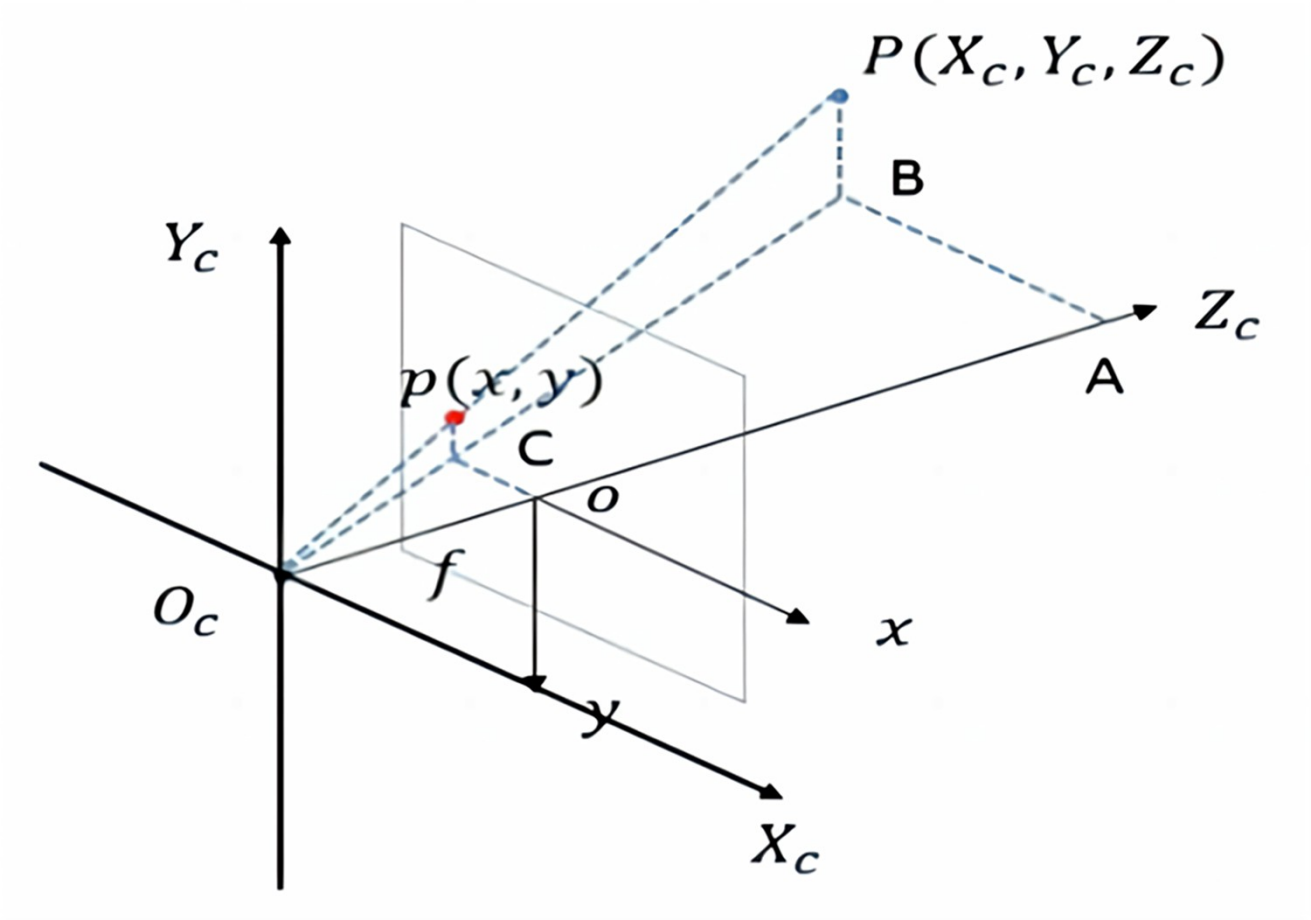
Coordinate conversion relationship between camera and image.

Establishing accurate coordinate transformations among the lidar, three-dimensional world, camera, image, and pixel coordinate systems is crucial for the successful integration of lidar and vision. The fusion process between lidar and visual sensors primarily involves translating measurements from diverse sensor coordinate systems into a standardized one. Fundamentally, sensor calibration overlays the perception outputs of the lidar onto the image. This procedure can be delineated into the subsequent three steps:

1. Transforming the world coordinate system into the camera coordinate system, the conversion relationship can be expressed as:
$$\left[\begin{array}{c} X_{c}\\ Y_{c}\\ Z_{c}\\ 1 \end{array}]=[\begin{array}{cc} R_{3\times 3} & T_{3\times 1}\\ 0_{1\times 3} & 1 \end{array}][\begin{array}{c} X\\ Y\\ Z\\ 1 \end{array}\right]$$ (2)
  Where *X*_*c*_, *Y*_*c*_, *Z*_*c*_ are the camera coordinates, *X*, *Y*, *Z* are the world coordinates of the lidar, *R*_*3x3*_ and *T*_*3x1*_ are the rotation and translation matrix of the camera respectively, and 0_1x3_ is a 1x3 zero matrix.
2. Transform the camera coordinate system into the image coordinate system (Fig 3), the conversion relationship can be expressed as:
$$Z_{c}[\begin{array}{l} x\\ y\\ 1 \end{array}]=[\begin{array}{cccc} f & 0 & 0 & 0\\ 0 & f & 0 & 0\\ 0 & 0 & 1 & 0 \end{array}][\begin{array}{c} X_{c}\\ Y_{c}\\ Z_{c}\\ 1 \end{array}]$$ (3)
  Among them, *x*, *y* are the camera coordinates, *f* is the camera focal length, and *X*_*c*_, *Y*_*c*_, *Z*_*c*_ are consistent with Formula (2).
3. Transform the image coordinate system into the pixel coordinate system, the conversion relationship can be expressed as:
$$Z_{c}[\begin{array}{l} u\\ v\\ 1 \end{array}]=[\begin{array}{ccc} \frac{1}{d_{x}} & 0 & u_{0}\\ 0 & \frac{1}{d_{y}} & v_{0}\\ 0 & 0 & 1 \end{array}][\begin{array}{cccc} f & 0 & 0 & 0\\ 0 & f & 0 & 0\\ 0 & 0 & 1 & 0 \end{array}][\begin{array}{cc} R_{3\times 3} & T\\ 0_{1\times 3} & 1 \end{array}][\begin{array}{c} X\\ Y\\ Z\\ 1 \end{array}]$$ (4)
  Where *u*, *v* are the pixel coordinates, *d*_*x*_, *d*_*y*_ are the length and width of a single pixel in the image plane, and *u*_*0*_, *v*_*0*_ are the coordinates of the origin of the image coordinate system in the pixel coordinate system.

## Fusion perception algorithm

**Fig 4:**
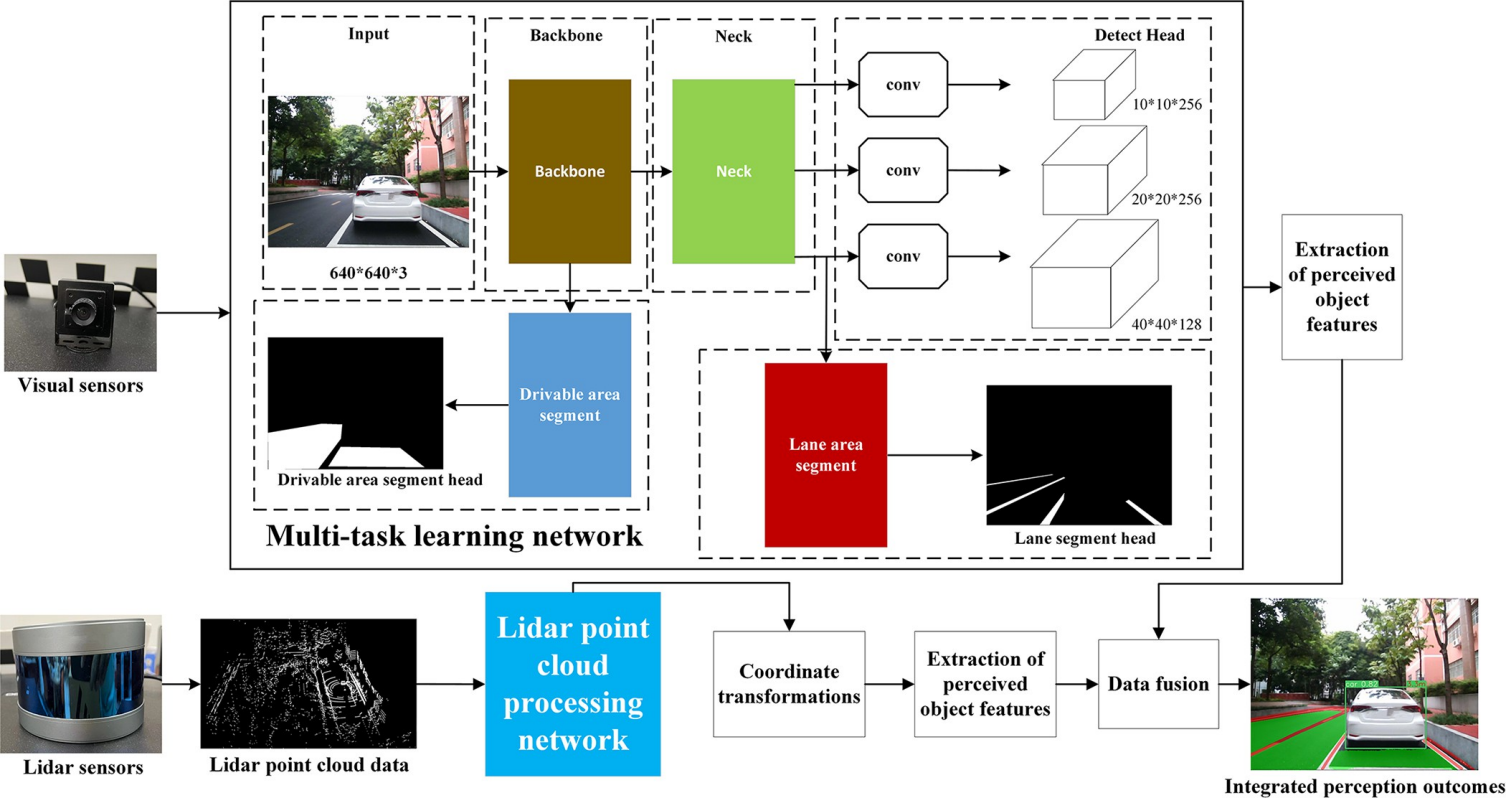
Network structure of perception fusion algorithm.

This paper proposes a multi-sensor fusion perception algorithm that is built upon the framework of the multi-task learning network. A backbone network is employed to detect three tasks: lane lines, vehicles, and drivable areas. And through the fusion of lidar and vision sensor data, the features of both are merged, resulting in a more comprehensive and informative representation. The architecture of the perception fusion network is illustrated in Fig 4. In this section, we present the designed multi-task learning network, the point cloud processing procedure of lidar, and the feature-level fusion strategy of multiple sensors, based on the relevant content mentioned above.

### Improved YOLOP algorithm

**Fig 5:**
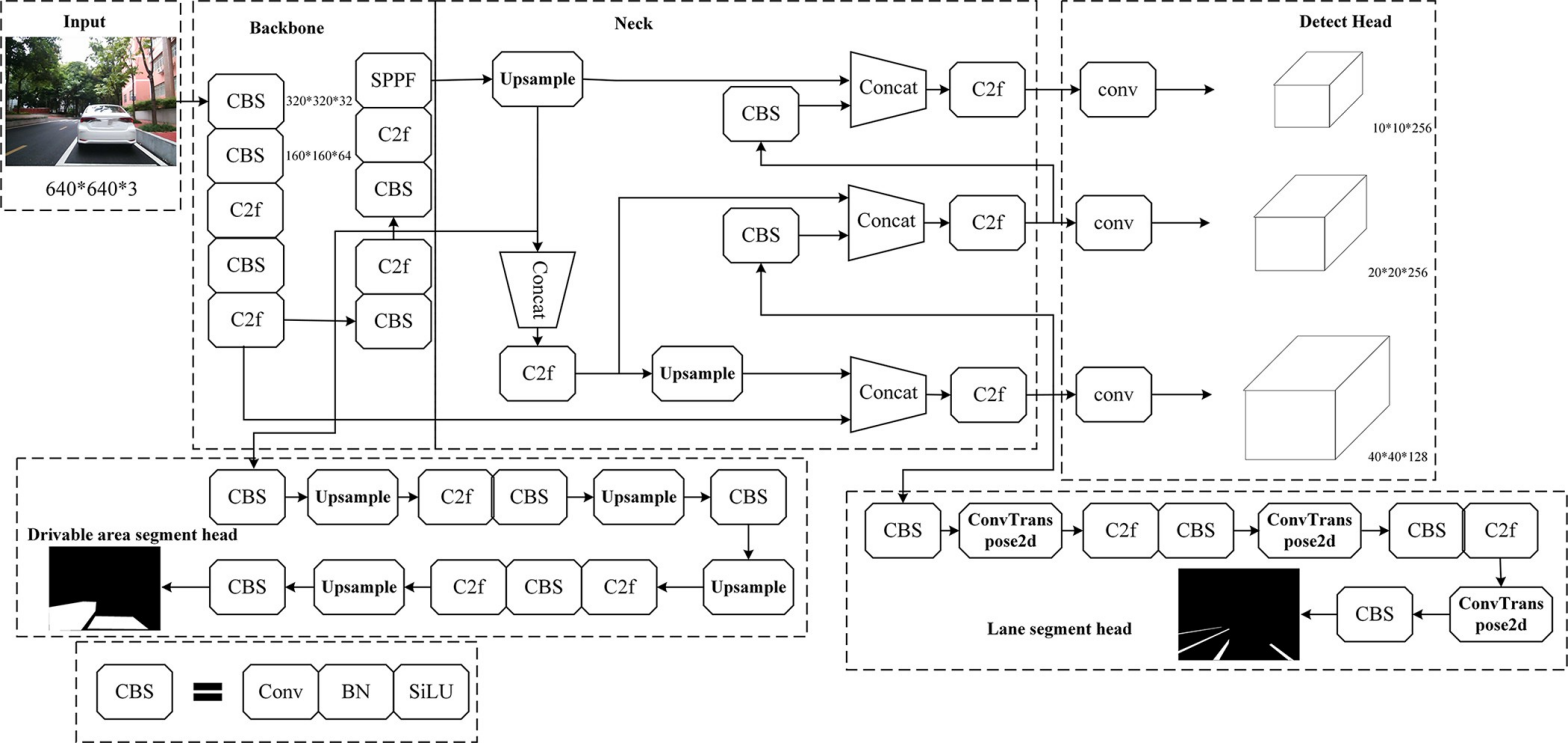
Network structure of multi-task learning.

The multi task learning algorithm in this paper introduces C2f structure on the basis of YOLOP network structure. The C2f structure aims to expedite both the model’s training process and inference speed. Additionally, the SPPF is incorporated to effectively address the adaptive issue encountered during the feature map pooling operation, while the ConvTranspose2D is introduced to tackle the problem of detail loss. Furthermore, in order to enhance the detection performance of multi-task learning, adjustments and optimizations are made to the feasible driving area and lane detection module. Specifically, an additional layer is added to the lane detection component, augmenting the network’s capacity for processing intricate information. The network structure can be visualized in Fig 5. This section outlines the designed multi-task learning network structure and elaborates on the specific optimizations and enhancements made to the original modules, network structure, and loss functions, all based on the YOLOP multi-task learning network.

### Improved module

**Fig 6:**
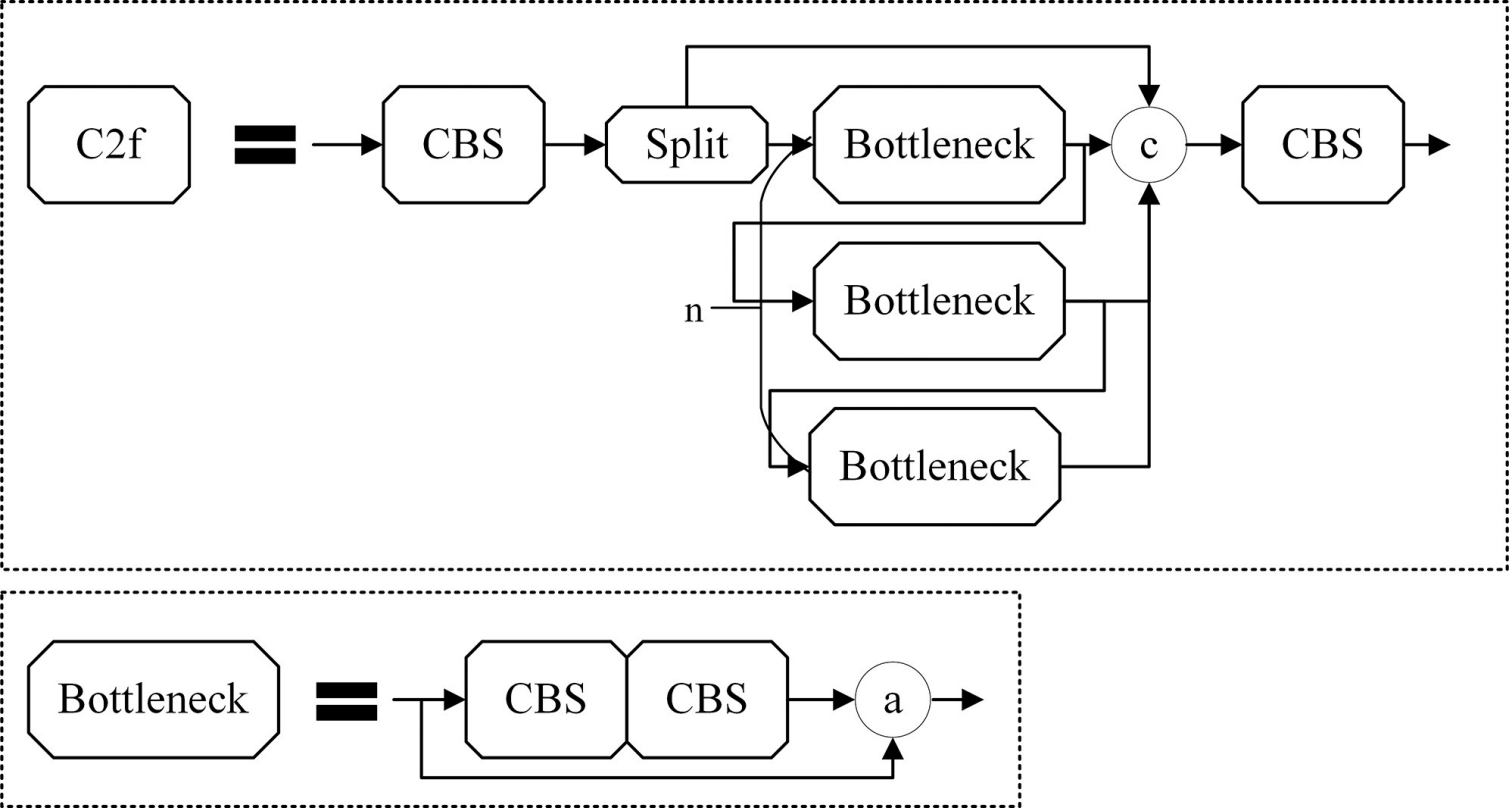
Structure of C2f.

*1*.*C2f module*. In our modified network architecture, the BottleneckCSP module is supplanted by the C2f module [23], as depicted in Fig 6. This alteration significantly curtails computational overhead by channel reduction, leading to expedited model training and inference. Moreover, it substantially mitigates GPU memory consumption. The C2f module stands out, restricting information flux, averting information degradation, and amplifying both robustness and the model’s generalization capability. Such attributes are invaluable for object detection in intricate settings. Refinements in network structures, coupled with the elimination of superfluous computations, further diminish model parameters and computational intricacy, all the while preserving model precision and efficacy.

**Fig 7:**
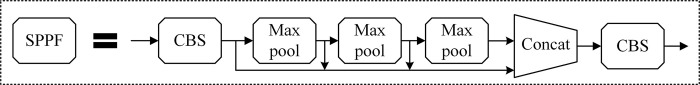
Structure of SPPF.

*2*.*SPPF module*. In our enhanced network, the SPP module is substituted by a more efficient SPPF module [24], illustrated in Fig 7. This module offers a tangible reduction in both computational requirements and storage demands. The SPPF module integrates convolutional strata with fully connected layers. When juxtaposed with the SPP module, it boasts a leaner parameter set and an accelerated computation rate. Additionally, it magnifies the model’s receptive ambit and expressiveness. The SPPF module adaptively conducts pooling of varying magnitudes on the feature map, ensuring extraction of multi-scale feature information. This adaptability circumvents issues associated with information omission or redundancy during drastic input image size variations. Furthermore, the SPPF module possesses a heightened ability for information amalgamation, adeptly integrating multi-scale data, subsequently enhancing model performance.

*3*. *ConvTranspose2D*. Within the lane line detection branch, Upsample is superseded by ConvTranspose2D [25]. Leveraging an adept convolution computation library, this module batch processes convolution operations on an array of input imagery, maximizing hardware capabilities like GPU for parallel computation, resulting in a marked improvement in processing speed. Contrary to Upsample, ConvTranspose2D consolidates both convolution and upsampling operations, streamlining the process. The Upsample method typically employs a static interpolation technique, devoid of the capability to glean the optimal transformation from datasets, rendering it less adaptable to specific tasks. Conversely, ConvTranspose2D, tailored to task-specific requirements, utilizes modifiable convolution kernel parameters, culminating in superior outcomes.

### Optimize network structure

*1*. *Introduce path aggregation network (PAN)*. ① To strengthen the information fusion and representation of different scale feature images, on the basis of the original FPN, add a PAN [26], which aligns the pixels in different scale feature images to make the number of pixels equal in different scale feature images, facilitating subsequent processing. FPN organically combines feature information of different scales, improves the representation ability of image features, and thus improves the recognition accuracy of the model.

② To further improve the efficiency and accuracy of object detection and segmentation tasks, add the drivable area detection module before FPN. This can preliminarily process the input image, remove some invalid areas, better integrate the information of the drivable area into the overall features, ensure the accuracy of the detection results, and increase the robustness of the model.

③ Connect the lane line detection module after FPN to avoid excessive computation of low-level features, which can improve computational efficiency and make the information of lane lines clearer after higher-level feature extraction and fusion, further improving the accuracy of lane line detection results.

*2*. *Improve network structure*. ① Enhancements to the model’s computational velocity are achieved by diminishing the convolution kernel’s size and stride. This approach reduces the feature map’s resolution and the convolution’s computational intricacy. In the head network segment, the number of convolutional, pooling, and deconvolution layers are minimized. Utilizing larger convolution and deconvolution kernels along with a deeper network structure amplifies the segmentation head’s resolution and precision on the feature map.

② Incorporating an additional upsampling layer within the drivable area detection branch transitions the architecture from the initial three-layer upsampling to a more refined four-layer structure. This modification effectively addresses the feature omission challenges engendered by superficial feature layers. Within the segmentation head network, a deconvolution layer of size 4 is integrated. By judiciously reducing model parameters, both the model’s speed and stability witness comprehensive improvements.

### Improve loss function

During the training process, adaptively adjust for different tasks and added datasets, and use different loss functions and training strategies, while summing the loss functions of all tasks with weights to achieve joint learning of multiple tasks. Such a design allows the algorithm to perform well in different scenes and datasets.

The total loss function is weighted and summed can be expressed as:

$$L_{sum}=\alpha_{1}L_{\det}+\alpha_{2}L_{da\_seg}+\alpha_{3}L_{lane\_seg}+\alpha_{4}L_{lane\_iou}$$ (5)

The total loss function, vehicle detection loss function, drivable area segmentation loss function, lane line segmentation loss function, and lane line detection loss function are represented by *L*_*sum*_, *L*_*det*_, *L*_*da_seg*_, *L*_*lane_seg*_, *L*_*lane_iou*_ respectively; *α*_*1*_, *α*_*2*_, *α*_*3*_, *α*_*4*_ are the weights of each loss function. Due to the structure of drivable areas and lane lines has been optimized and adjusted, leading to adjustments in *α*_*2*_ and *α*_*3*_ from the original value of 0.2 to 0.3 and 0.5, respectively, with the aim of enhancing attention to details. *α*_*1*_ and *α*_*4*_, on the other hand, remain unchanged and are set at 1 and 0.2, respectively.

The weighted sum of the loss function for vehicle detection can be expressed as:

$$L_{\det}=\alpha_{5}L_{box}+\alpha_{6}L_{obj}+\alpha_{7}L_{cls}$$ (6)

In the equation, *L*_*box*_, *L*_*obj*_, and *L*_*cls*_ represent the bounding box regression loss function, object confidence loss function, and classification loss function respectively; *α*_*5*_, *α*_*6*_, *α*_*7*_ are the weights of each loss function. In order to ensure consistency with the weight settings of the YOLOP network, the weights are specifically defined as 0.05, 1.0, and 0.5, respectively. To improve the effectiveness of vehicle detection, the loss functions *L*_*obj*_ and *L*_*cls*_ incorporate the utilization of the Focal loss.

Focal Loss can effectively alleviate the class balance problem between fewer class samples (vehicles, drivable areas, and lane lines) and a large number of background samples. Unlike traditional losses, Focal Loss assigns higher weights to samples that are difficult to classify and misclassified, effectively improving the network’s robustness to difficult samples and noisy data.

The Focal loss function [27, 28] can be expressed as:

$$L_{Focal}=FL(p_{t})=-\alpha_{8}{\left(1-p_{t}\right)}^{\gamma }\;\ln(p_{t})$$ (7)

In the equation, *α*_*8*_ is a balancing factor used to solve the problem of imbalance between positive and negative samples; *γ* is an adjustment factor used to adjust the weights of easy and difficult samples; *p*_*t*_ is the predicted probability output by the network, and *ln(p*_*t*_*)* is the logarithm of the predicted value. During the training phase of the model, the parameter *α*_*8*_ is assigned a value of 0.25, which allows for the adjustment of the weight assigned to negative samples to be four times greater than that assigned to positive samples. This adjustment prevents the model from overly prioritizing the more prevalent negative samples. Additionally, the parameter *γ* is set to 2 in order to amplify the weight of challenging samples. This amplification facilitates the model’s focus on samples that are difficult to classify, ultimately leading to an enhancement in the performance of the model.

Lane line detection has a certain degree of difficulty in multi-task learning networks because the shapes and colors of lane lines vary greatly, and they are often obscured. Before adopting the Dice Loss, the CrossEntropy Loss (CE Loss) was used as the loss function for the lane line detection task, and lane line detection was achieved through the method of positive sample matching. However, this method has a significant problem: the ratio of the number of lane line pixels to background pixels is extremely imbalanced, which makes the model more inclined to predict background pixels and ignore lane line pixels.

Dice Loss calculates the similarity between the predicted result and the true value on a per-pixel basis [29, 30], which can effectively solve the problem of pixel number imbalance. The loss function can be expressed as:

$$L_{Dice}=Dice(y,\overset{\wedge }{y})=\frac{2|y\cap \overset{\wedge }{y}|}{\left|y|+|\overset{\wedge }{y}\right|}$$ (8)

In the equation, *y* and $\overset{\wedge }{y}$ respectively represent the authentic labels and the labels as forecasted by the model. Using Dice Loss as the loss function can effectively measure the degree of match between the model’s predicted results and the actual values, and make the model pay more attention to the detection of lane line pixels. Leveraging Focal Loss can further ameliorate the issue of an excessive number of negative samples in lane line detection. This enhancement heightens the network’s attention to the lane lines, thereby augmenting the accuracy of lane line detection.

To further optimize the performance of lane line detection in multi-task learning networks, a combination loss function combining Focal Loss and Dice Loss [31, 32] is used for model training. Dice Loss and Focal Loss respectively target the influence of factors such as diverse lane line shapes, colors, and frequent obscuration, emphasize the detection of lane line pixels, and improve the model’s attention to lane lines, while optimizing the problem of a large number of background pixels in the training dataset. The combination of these two loss functions can fully utilize their complementary advantages, effectively improving the accuracy and robustness of the lane line detection task.

The combined loss function of focal loss and dice loss can be expressed as:

$$L_{lane\_seg}=L_{Focal}+\lambda L_{Dice}$$ (9)

In the equation, *λ* is a constant that controls the weight ratio of Dice loss in the lane line loss function. To enhance the model’s focus on the accuracy of lane lines and the overlap of areas, while paying relatively less attention to class imbalance, the parameter *λ* is set to 2. By increasing the weight ratio of the Dice Loss, the model can make more precise predictions of lane lines, consequently improving the overall performance.

### Point cloud processing algorithm

**Fig 8:**
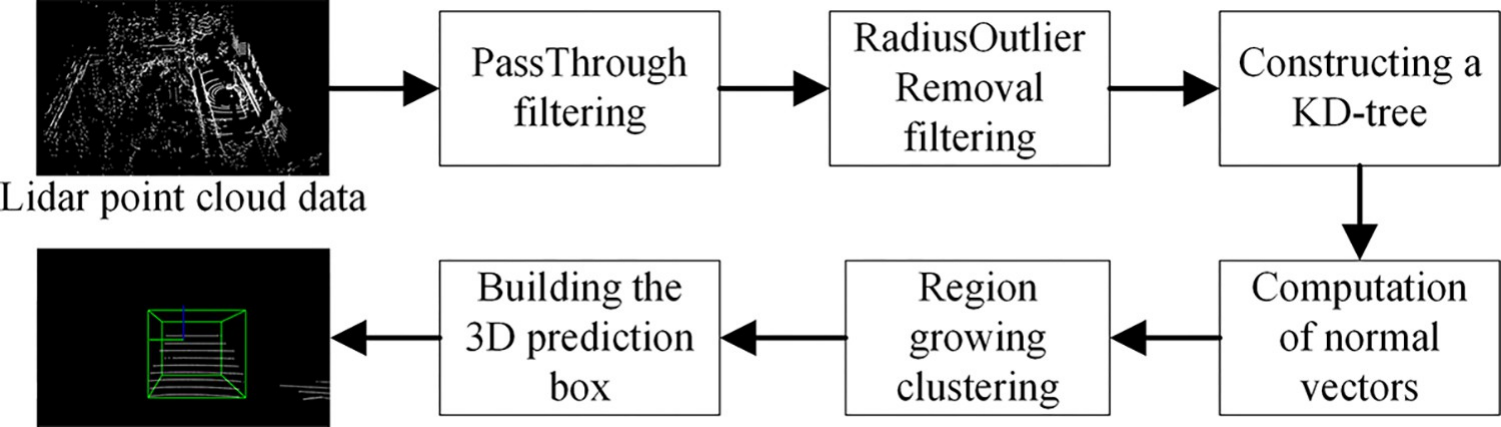
Lidar point cloud processing process.

To provide more accurate and reliable scene understanding and perception results, the original laser point cloud data is processed in a series. The processing process is shown in Fig 8. This section introduces the technical approach used in processing lidar point clouds.

### PassThrough filtering

To improve the processing effect on point cloud data and reduce the complexity of subsequent point cloud data processing, PassThrough filtering [33] is introduced as a preprocessing step for laser point cloud. PassThrough filtering is a preprocessing method used for laser point cloud processing, aiming to remove invalid data in the vertical or horizontal direction, and is suitable for tasks of extracting specific attribute areas in the point cloud. For example, the perception range around the vehicle body can be extracted by setting a distance range.

### Radius outlier removal (ROR) filtering

During the driving process of a car, the onboard sensors may be affected by various factors such as weather conditions, obstructions, or sensor failures, causing the panoramic driving system’s perception performance to decline. Therefore, ROR filtering [34] can remove abnormal interference points and maintain local structural information of point cloud data, such as vehicle shape or road contour. ROR filtering can also reduce the interference of noise and stray points in point cloud data on obstacle detection and scene understanding, thereby improving the accuracy of the perception system for obstacles and enhancing driving safety performance.

### Region growing clustering

In autonomous driving, the car driving system needs to understand the complex road environment in which the vehicle is located, including elements such as lane lines, traffic signs, and pedestrians. In order to achieve a more comprehensive and accurate understanding of the driving environment, key scene elements are identified and extracted through the region growing clustering algorithm [35], and obstacles are segmented based on the local information and features of the point cloud, clustering adjacent points together to form accurate obstacle boundaries, thereby achieving more reliable obstacle perception and tracking. The region growing clustering algorithm can adaptively determine the parameters of clustering, such as the minimum or maximum clustering range, the number of neighborhood points. This enables the algorithm to produce satisfactory results across various scenes and datasets.

### Fusion algorithm

**Fig 9:**
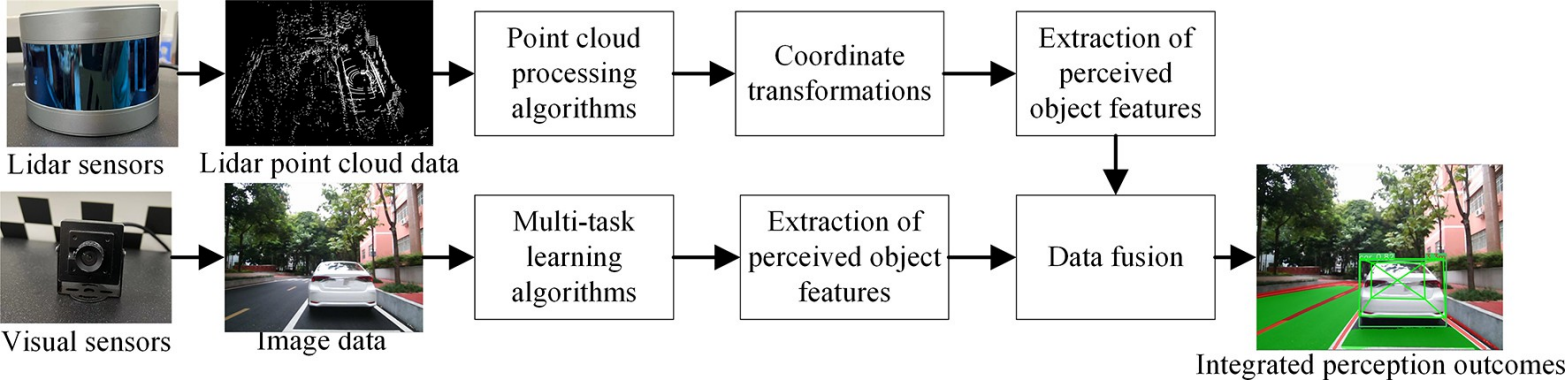
Flow chart of fusion perception.

The fusion perception algorithm uses the high-precision distance data provided by lidar to identify and locate potential obstacles, providing an important reference for the vehicle’s obstacle avoidance decision-making. At the same time, the algorithm can accurately distinguish different categories of objects, such as vehicles, pedestrians, or traffic signs, and assign them semantic labels. In addition, the fusion of visual information and lidar distance information can provide the vehicle with accurate lane line positioning and trajectory tracking results, and can accurately detect the vehicle’s drivable area, enhancing the vehicle’s understanding and planning ability of the road environment. Such a feature-level fusion strategy provides more comprehensive and accurate information, enhancing the performance of the panoramic driving perception system and the reliability of decision-making. The fusion perception process is shown in Fig 9. This section introduces the strategy design for feature-level fusion of lidar and visual sensor data.

## Experiment

In order to verify the effectiveness and reliability of the perception fusion algorithm proposed in this paper, this section not only trains the multi-task learning network model but also compares the training results with different networks (including CNN and transformer models) and different datasets (BDD100K and KITTI) to highlight the superiority and generalizability of the multi-task network. In addition, the feasibility and reliability of the multi-sensor fusion strategy are validated, and an analysis of the final visualization results is conducted.

### Experimental setup

The experiments are all implemented in an environment with an RTX3090 24G GPU, using the PyTorch 1.9.0 deep learning framework. The experiments involved in this study were conducted using the publicly available BDD100K dataset. This dataset consists of 100,000 images of driving routes covering approximately 100,000 kilometers. The dataset was divided into training, testing, and validation sets using a 7:2:1 ratio to ensure the experiment’s results are reliable and reproducible. During the model training process, a cosine annealing strategy is used to adjust the learning rate, the number of training iterations is set to 200, the batch size is set to 32, and the time for one iteration is about 32 minutes. The first three training cycles are set as warm-up training to further optimize the model performance during the training process.

The performance assessment of the experimental results mainly includes mAP50, Recall, mIoU, Accuracy, and IoU. Among them, mAP50 and Recall are the evaluation metrics for vehicle detection, mIoU is for drivable area detection, and Accuracy and IoU are for lane line detection. Their calculation formulas are can be expressed as:

$$mAP50=AP50=\sum_{n}(R_{n}-R_{n-1})P_{n}(IoU=0.5)$$ (10)

$$Recall=\frac{TP}{TP+FN}$$ (11)

$$mIoU=\frac{1}{k+1}\sum_{i=0}^{k}\frac{TP}{FN+FP+TP}$$ (12)

$$Accuracy=\frac{TP+TN}{TP+TN+FP+FN}$$ (13)

$$IoU=\frac{TP}{FN+FP+TP}$$ (14)

Among them, *P*_*n*_ and *R*_*n*_ denote the precision and recall at the nth threshold respectively. *R*_*n*_ and *R*_*n-1*_ correspond to two contiguous yet distinct intervals on the abscissa. *TP* (True Positive) refers to the quantity of pixels predicted as positive samples, which coincide with the actual annotations. *FN* (False Negative) pertains to the count of pixels predicted as negative samples that nonetheless overlap with the actual annotations. *FP* (False Positive) signifies the number of pixels predicted as positive samples yet bear no overlap with the actual annotations. *k* represents the quantity of samples. Lastly, *TN* (True Negative) refers to the number of pixels that are predicted as negative samples and align with the actual annotations.

### Analysis of model training results

#### Model performance analysis

The experiment compares the performance of four different networks in multitask processing, as illustrated in Table 1. Among these networks, the proposed multitask network significantly reduces the parameter count compared to HybridNets and YOLOPv2 networks, differing by only 3.1M parameters when compared to the YOLOP network. However, the improved network surpasses the YOLOP network in various key metrics, including recall, mean Intersection over Union (mIoU), and accuracy. Additionally, the performance of this approach exceeds that of both the transformer-based model proposed by Wenjie Zhu and the YOLO-ODL model, and the improve network exhibits superior speed in comparison to both the HybridNets and YOLOPv2 networks while performing on par with the YOLOP network. This facilitates more precise and efficient handling of multiple tasks while utilizing fewer computing resources. Moreover, it showcases tremendous potential and competitiveness in practical application scenarios.

**Table 1:** Experimental results of different network performance.

Network	Params	mAP50 (%)	Recall (%)	mIoU (%)	Accuracy (%)	IoU (%)	FPS (inf+nms)
YOLOP [12]	7.9M	76.5	89.2	91.5	70.5	26.2	17.4ms
HybridNets [13]	12.8M	77.3	92.8	90.5	85.4	31.6	61.4ms
YOLOPv2 [14]	38.9M	83.4	91.1	93.2	87.3	27.2	20.6ms
Wenjie Zhu [36]	8.3M	75.8	89.1	91.9	74.9	27.7	-
YOLO-ODL [37]	22.2M	79.7	94.2	92.3	75.0	27.5	-
Ours	11.0M	80.2.	91.3	93.6	82.1	27.5	18.0ms

### Analysis of vehicle detection results

The BDD100K dataset includes many traffic objects, such as buses, trucks, trains., which have similarity and correlation with cars in terms of shape, size. To increase the diversity of training data, these traffic objects are merged into a single car category when processing the dataset, to improve the accuracy and robustness of vehicle detection. The improved network is compared with traditional multitasking and single-task detection networks, as shown in Table 2.

**Table 2:** Experimental results of vehicle detection (* multi-task).

Network	mAP50(%)	Recall(%)
MultiNet* [38]	60.2	81.3
DLT-Net* [39]	68.4	89.4
Faster R-CNN [40]	55.6	77.2
YOLOv5s [8]	77.2	86.8
YOLOP* [12]	76.5	89.2
HybridNets* [13]	77.3	92.8
YOLOPv2* [14]	83.4	91.1
Wenjie zhu* [36]	75.8	89.1
EHSINet* [41]	79.3	91.3
YOLO-ODL* [37]	79.7	94.2
Ours*	80.2	91.3

The results show that the networks based on the YOLO series perform well in vehicle detection. The improved network improves the mAP50 indicator by 20.0% compared to the traditional multi-task detection network MultiNet, and increases by 3.0% compared to the single-task network YOLOv5s, and it also surpasses the transformer model by Wenjie Zhu with a 4.4% improvement. Although it is slightly reduced compared to YOLOPv2 in terms of mAP50, the overall performance is still excellent, surpassing most vehicle detection networks.

### Analysis of driveable area detection results

The comparison of the drivable area detection network experiment results is shown in Table 3. The results show that networks based on the YOLO series, such as YOLOP, HybridNets, and YOLOPv2, perform well on the BDD100K dataset. Compared to other networks, the proposed multi-task network exhibits outstanding performance in the drivable area detection task, surpassing the multi-task network DLT-Net by 22.3% and the single-task network PSPNet by 4%, it surpasses the performance of Team Host_29005 on the BDD100K challenge website by a remarkable margin of 10%. Moreover, improved by 6.2% compared to the transformer-based multi-task model by Xiwen Liang, thereby showcasing exceptional detection capabilities. In conclusion, the enhanced network demonstrates outstanding performance in the drivable area detection task.

**Table 3:** Experimental results of drivable area detection (* multi-task).

Network	mIoU(%)
MultiNet* [38]	71.60
DLT-Net* [39]	71.30
PSPNet [42]	89.60
YOLOP* [12]	91.50
HybridNets* [13]	90.50
YOLOPv2* [14]	93.20
Yue Yu [43]	90.19
Wenjie Zhu* [36]	91.90
EHSINet* [41]	92.30
YOLO-ODL* [37]	92.30
Xiwen Liang* [44]	87.40
Host_29005_Team [45]	83.65
Ours*	93.60

### Analysis of lane line detection results

The comparison of lane line detection experiment results is shown in Table 4. The results show that compared to other networks (such as ENet, SCNN, ENet-SAD, YOLOP.), the improved network performs better, especially compared to the single-task network ENet, the performance is improved by nearly 48%. In comparison to Wenjie Zhu’s multi-task Transformer model, the performance improvement was 7.2%. Despite the slight decrease in our model’s performance, as presented in this paper, when compared to the HybridNets and YOLOPv2 models, it still showcases certain performance advantages. Notably, our model’s parameter count is reduced by 1.8M and 27.9M in comparison to HybridNets and YOLOPv2 models, respectively. Additionally, our model achieves higher FPS (frames per second) than both of them. Therefore, we can infer that the improved network is relatively small and has excellent performance in lane line detection. It can implement localized processing on some edge devices and show good accuracy and detail capture ability.

**Table 4:** Experimental results of lane detection (* multi-task).

Network	Accuracy(%)	IoU(%)
Enet [2]	34.12	14.64
SCNN [46]	35.79	15.84
ENet-SAD [47]	36.56	16.02
YOLOP* [12]	70.50	26.20
HybridNets* [13]	85.40	31.60
YOLOPv2* [14]	87.31	27.25
Wenjie Zhu* [36]	74.90	27.70
EHSINet* [41]	79.40	27.40
YOLO-ODL* [37]	75.00	27.50
Ours*	82.10	27.50

### Ablation experiment

Based on the YOLOP network, the network performance is improved through the improvement and optimization of the network structure, hyperparameters, and loss functions. At the same time, through quantitative and qualitative comparison experiments, the improved multi-task network has significantly improved in various performance indicators compared to the YOLOP network. The comparison of ablation experiment results is shown in Table 5. During the network training process, the effects of different training methods and loss functions on network performance are fully considered.

**Table 5:** Ablation experimental results.

Training method	mAP50(%)	Recall(%)	mIoU(%)	Accuracy(%)	IoU(%)
YOLOP(Baseline)	76.5	89.2	91.5	70.5	26.2
Backbone+Fine-tuned	78.1	89.6	92.0	75.4	27.6
Dice loss	77.8	89.1	92.3	79.4	27.1
Focal loss+Dice loss	79.8	90.7	92.8	81.5	27.6
Convtranspose2d	80.2	91.3	93.6	82.1	27.5

### Analysis of training results on the KITTI dataset

To assess the generalization capability of the proposed multi-task learning network model, we conducted experiments using the KITTI dataset. Given that the KITTI dataset solely provides object detection data and lacks drivable areas and lane line datasets, our focus was exclusively on validating the object detection aspect of the multi-task learning network. The dataset comprises 7,481 images, and the data was partitioned in a ratio of 7.5:1.5. Only the data partition ratio was varied in the experiment, while maintaining consistent settings with the aforementioned experiments. The experiment results are presented in Table 6.

**Table 6:** Traffic object detection results: Comparing on the KITTI dataset.

Network	Params	Prediction(%)	Recall(%)	mAP50(%)	Time/epoch (s)
YOLOP [12]	6.8M	91.5	81.9	91.2	138.0
Ours	4.7M	93.2	87.2	94.6	36.0

According to the experimental results in Table 6, the multi-task learning network proposed in this paper demonstrates superior performance in object detection compared to YOLOP. The parameter count is reduced by 2.1M, while the model training time is reduced by 3.8-fold. Overall, the evaluation metrics surpass those of the YOLOP model. Consequently, the training results on the KITTI dataset robustly validate the generalization capability and superiority of the proposed multi-task learning network.

### Joint calibration of lidar and vision

#### Vision sensor calibration

**Fig 10:**
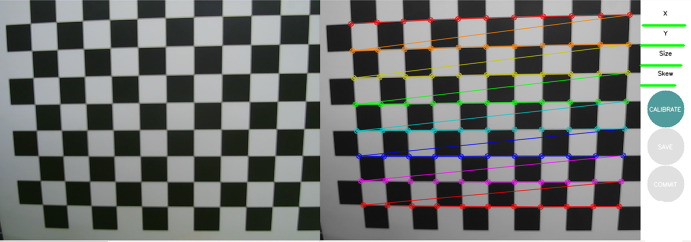
Calibration process of vision sensor.

The vision sensor used in the experiment is a 640x480 pixel USB camera, and the internal and external parameters of the camera are obtained using the camera calibration tool in Autoware, as shown in Fig 10.

Among them, X represents the situation of left and right movement in the field of view, Y represents the situation of up and down movement, Size represents the situation of the field of view being full, and Skew represents the situation of angle change. When the progress bar turns green and is full, the calibration is completed. Finally, the internal parameters and distortion data of the camera are calculated. The final internal parameter matrix A and distortion parameters B are as follows:

$$\begin{array}{l} A=\left[\begin{array}{ccc} 441.4963 & 0 & 323.7187\\ 0 & 430.3780 & 238.1937\\ 0 & 0 & 1 \end{array}\right]\\ B=[\begin{array}{ccccc} 0.2303 & -0.9156 & -0.0104 & 0.0009 & 1.3507 \end{array}] \end{array}$$

### Joint calibration of lidar and vision

**Fig 11:**
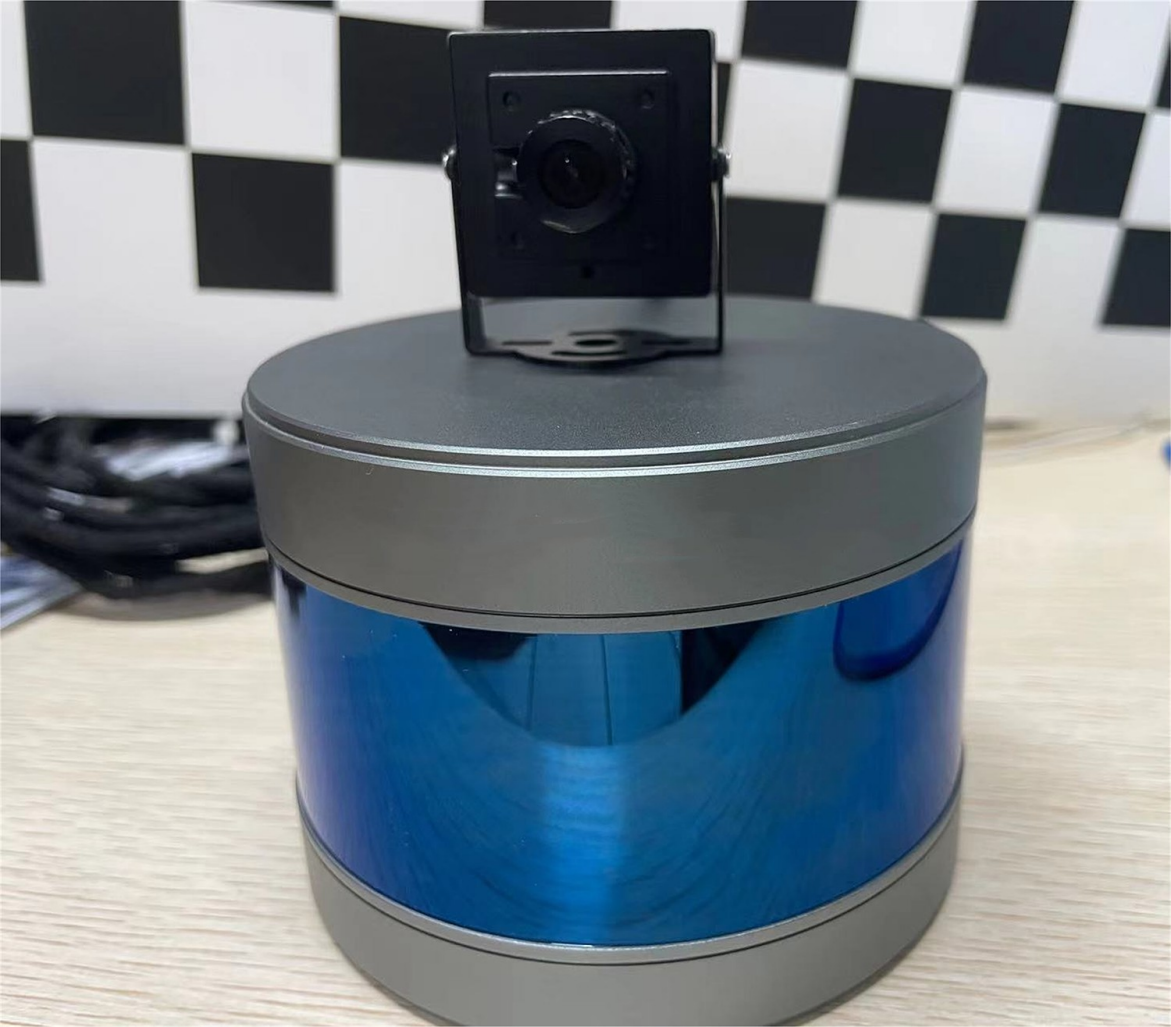
Lidar and vision combination platform.

The lidar used in the experiment is a robosense 16-line hybrid solid-state lidar with a measuring distance of up to 150 meters, a horizontal measuring angle of 360°, up to 300000 points per second, and a vertical measuring angle of -15°-15°. The combination platform of laser and vision is about 1 meter above the ground, as shown in Fig 11.

Before joint calibration, a point cloud packet of the calibration board at different positions needs to be recorded, and by replaying the recorded point cloud packet, 9 different pixel point cloud pairs are selected. These data are used to obtain the external parameter matrix of the combination platform, namely the rotation matrix and the translation matrix. The final external parameter matrix C is as follows:

$$C=[\begin{array}{cccc} 0.0585 & 0.0792 & 0.9951 & -0.0483\\ -0.9983 & 0.0032 & 0.0585 & -0.0317\\ 0.0015 & -0.9969 & 0.0792 & 0.0328\\ 0 & 0 & 0 & 1 \end{array}]$$

### Processing of lidar point cloud

#### PassThrough filtering

**Fig 12:**
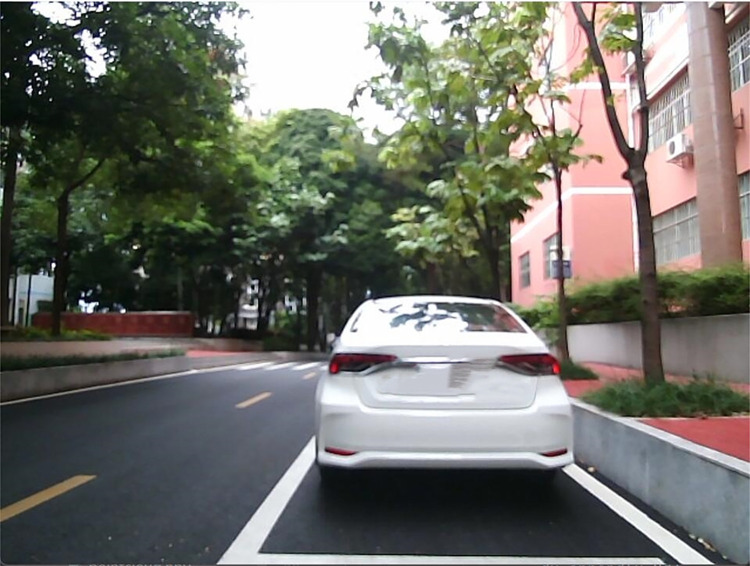
Camera view.

**Fig 13:**
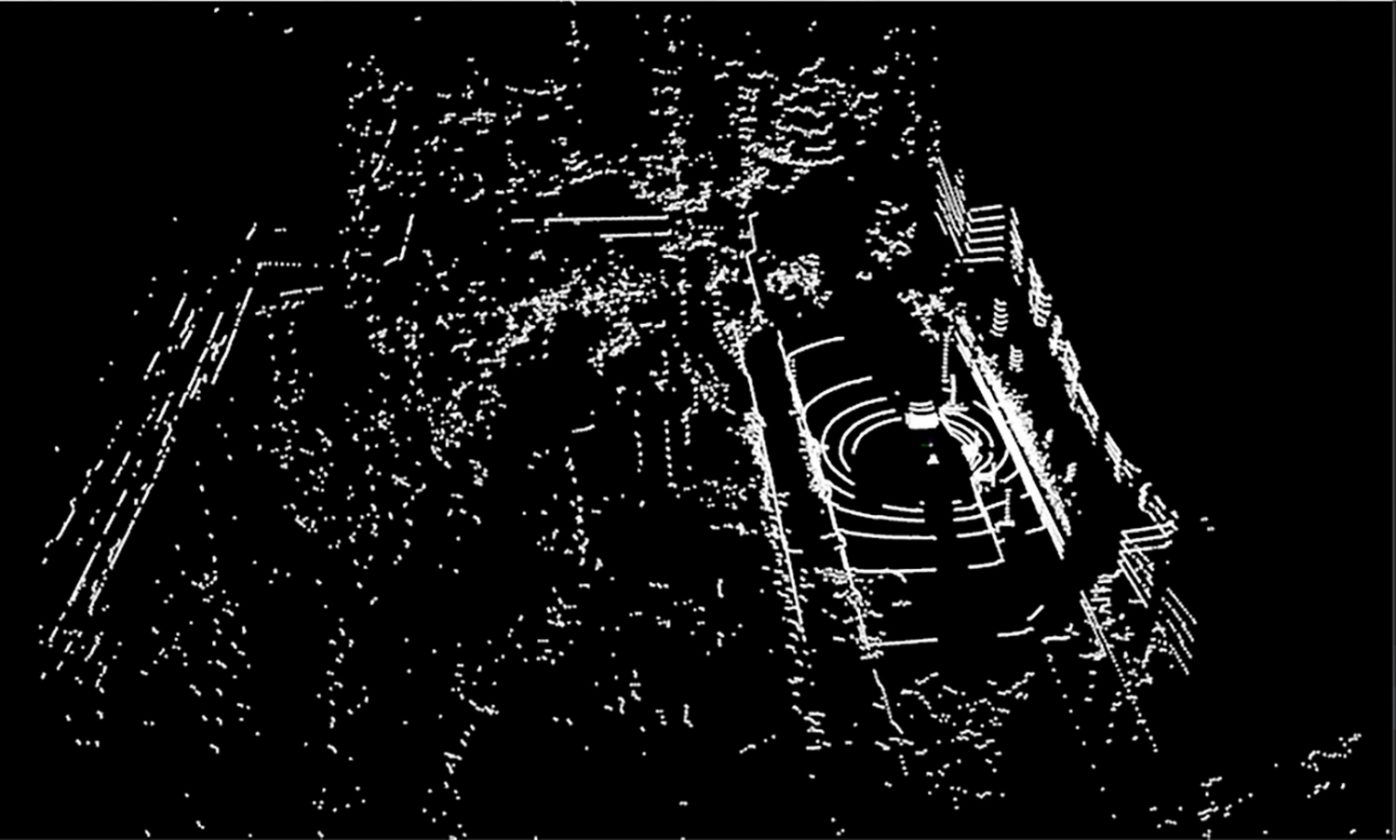
Original point cloud.

To be consistent with the view in front of the car, as shown in Fig 12, the PassThrough filter is used to limit the original point cloud (as shown in Fig 13) to a forward distance of 0.3–20 meters. At the same time, to avoid the interference of ground point clouds, the original point cloud is limited to a height of -0.9–5 meters. The number of point clouds is reduced from the original 28800 to 9124.

#### ROR filtering

To facilitate the construction of the KD tree and reduce the computational load of the algorithm, the experiment sets the ROR filter search radius to 0.1 and sets a point cloud to have at least 10 neighboring points within this radius to be retained. It can filter out most of the interference from environmental factors.

#### Region growing clustering

**Fig 14:**
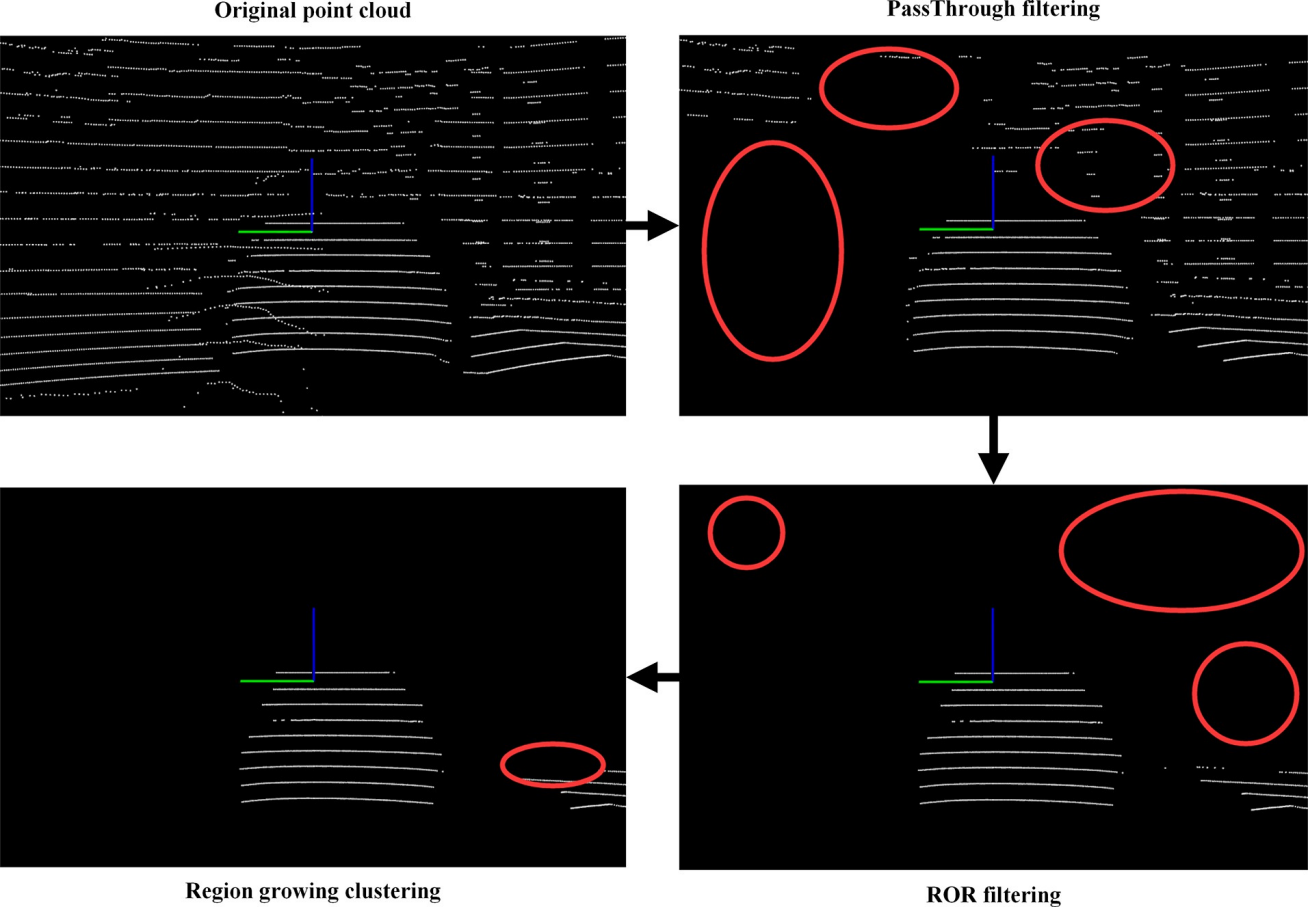
Lidar point cloud processing results.

The experiment obtains the geometric and surface feature information in the point cloud by constructing a KD-tree and calculating the normals, and uses the normal information for region growing clustering. In the experiment, the minimum and maximum cluster sizes are set to 30 and 10000 respectively, the number of neighboring searches is set to 20, the smoothness threshold is set to 70, and the curvature threshold is set to 1.0. The number of point clouds after clustering is 2648. All the above parameter settings are verified by comparative experiments. The lidar point cloud processing results as shown in Fig 14.

#### Construction of three-dimensional(3D) prediction boxes

**Fig 15:**
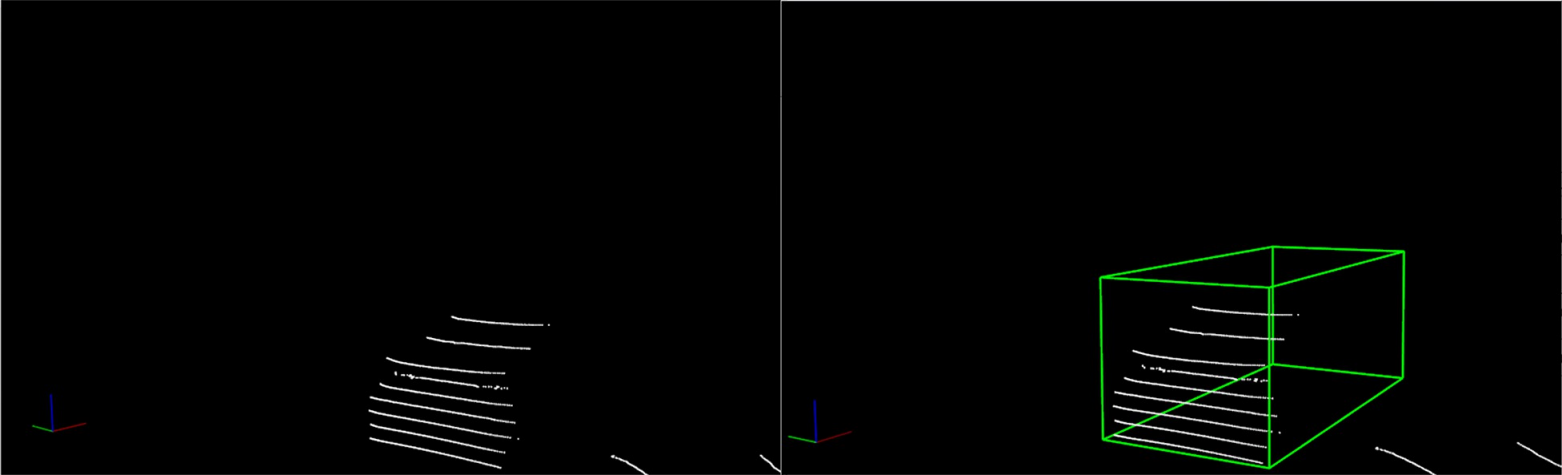
Construction of 3D prediction boxes.

The construction of 3D prediction boxes is a fundamental task in panoramic driving perception, as it offers crucial input for key tasks, including object detection, tracking, and decision-making. In this experiment, objects within the laser point cloud are segmented into distinct regions, and relevant features like geometric properties and point cloud density are extracted from each region. Utilizing these features, three-dimensional bounding boxes are constructed to precisely depict information such as the object’s position, size, and orientation. The results of this construction process are displayed in Fig 15.

#### Fusion of lidar and vision

**Fig 16:**
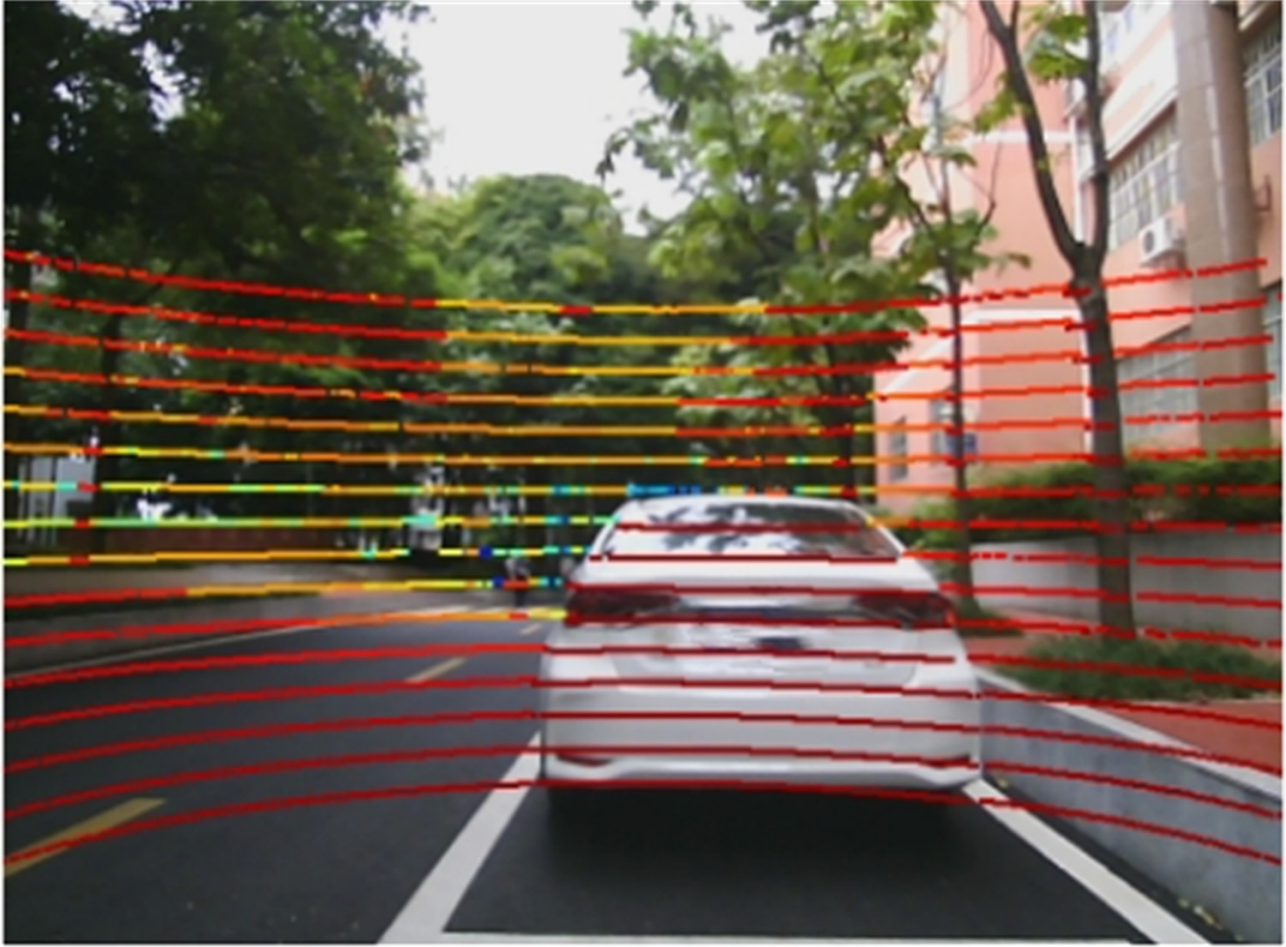
Preliminary fusion results.

Based on the data obtained from previous experiments, a preliminary fusion of lidar and vision is performed, i.e., the lidar point cloud coordinates are converted into pixel coordinates, as shown in Fig 16, ensuring that the point cloud and image have a consistent coordinate system.

**Fig 17:**
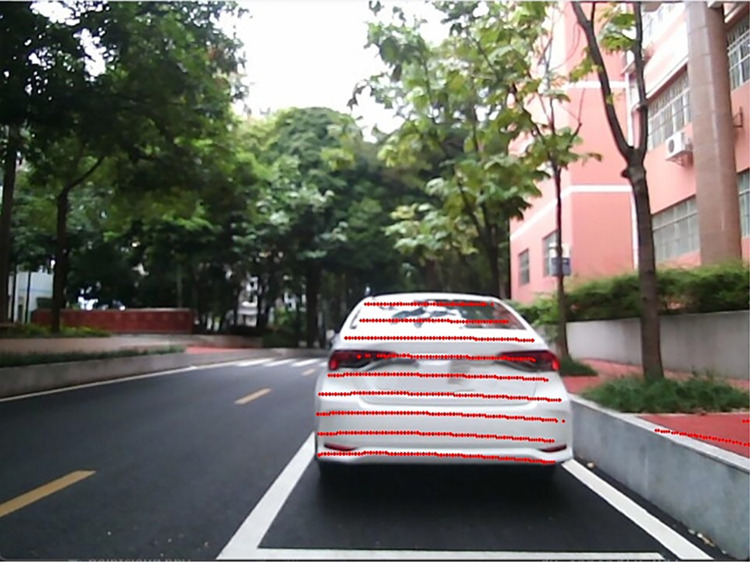
Point cloud processing results.

**Fig 18:**
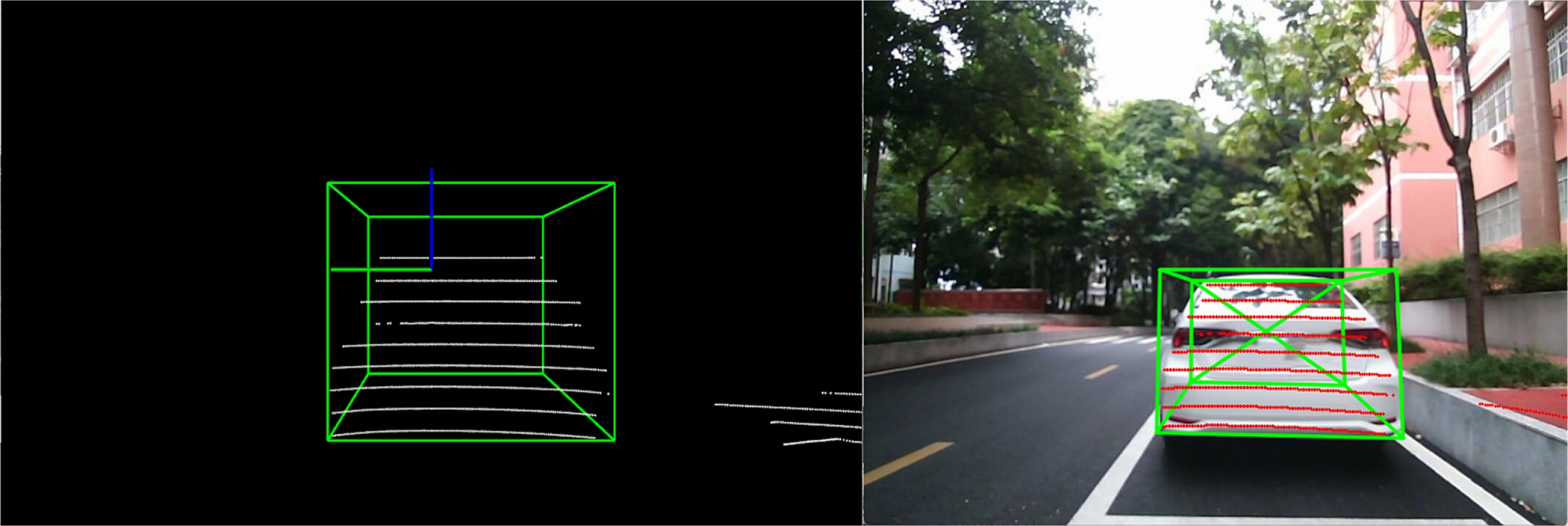
Fusion results of 3D prediction boxes.

Through a series of processes such as point cloud filtering and clustering, features of perceived objects can be extracted, including important information such as the position and actual distance of the object. The point cloud processing results are shown in Fig 17. The 3D prediction boxes of the objects are then fused with vision to obtain more accurate information about the object’s size and position. The results of this fusion are shown in Fig 18.

### Analysis of visualization results

#### Analysis of vision perception visualization results

**Fig 19:**
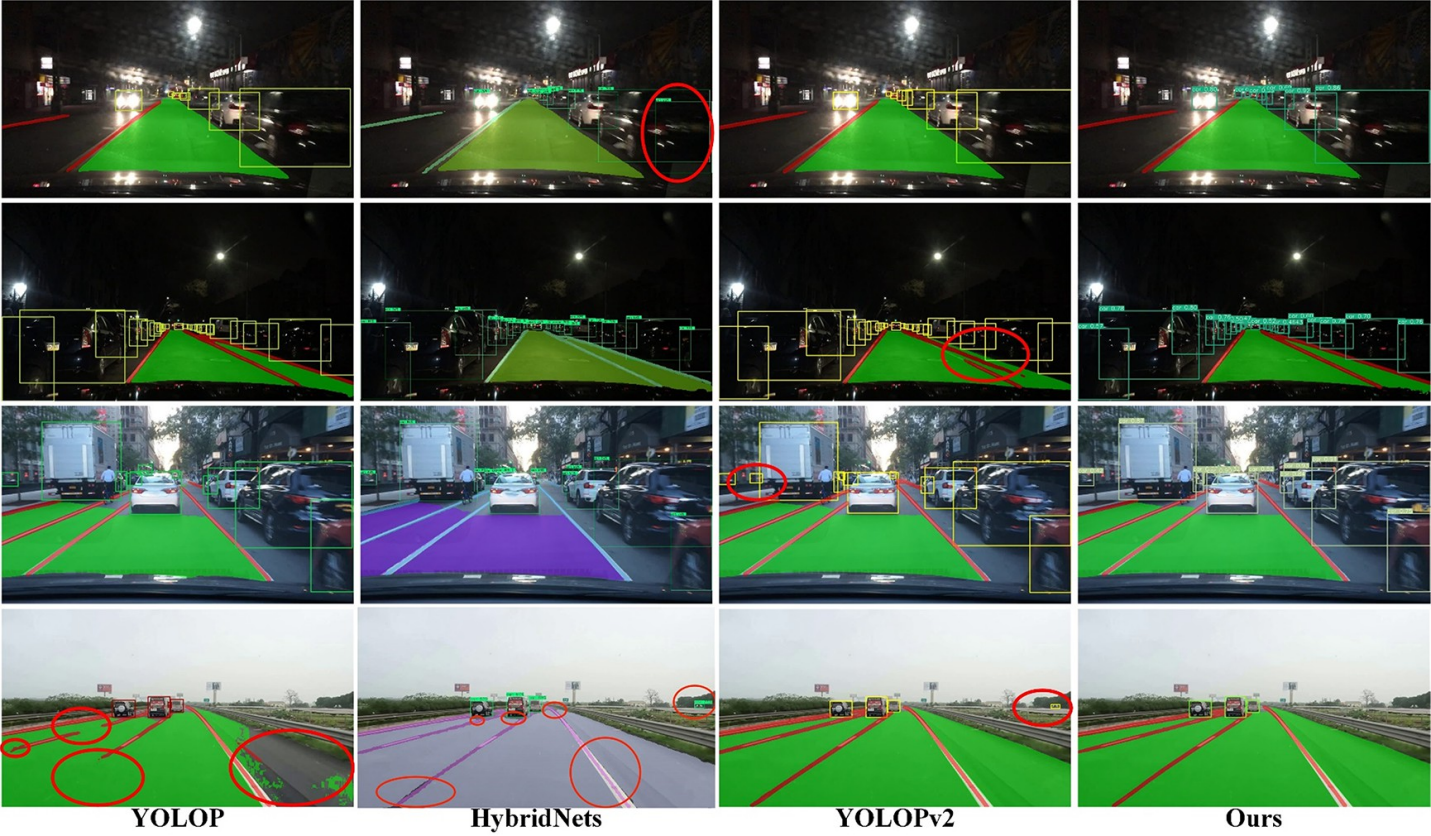
Comparison of visualization results.

The improved YOLOP model was compared with several state-of-the-art panoramic driving perception technologies. A unified confidence threshold of 0.25 and an IoU threshold of 0.45 were used to filter out inaccurate predicted boxes, ensuring high-quality detection objects. The experiments validated the effectiveness of the improved model in different environments and clarity scenes. Fig 19 presents a visual comparison of the experimental results. From the comparison in Fig 19, it is evident that the improved model outperforms the YOLOP and HybridNets models in terms of lane line and drivable area detection. It also exhibits superior robustness in lane line detection compared to YOLOPv2. In terms of vehicle detection, the HybridNets and YOLOPv2 models display a higher false positive rate based on the visual results. Thus, it can be concluded that the improved model outperforms the majority of existing models in terms of performance, while maintaining high robustness and accuracy.

#### Analysis of fusion perception visualization results

**Fig 20:**
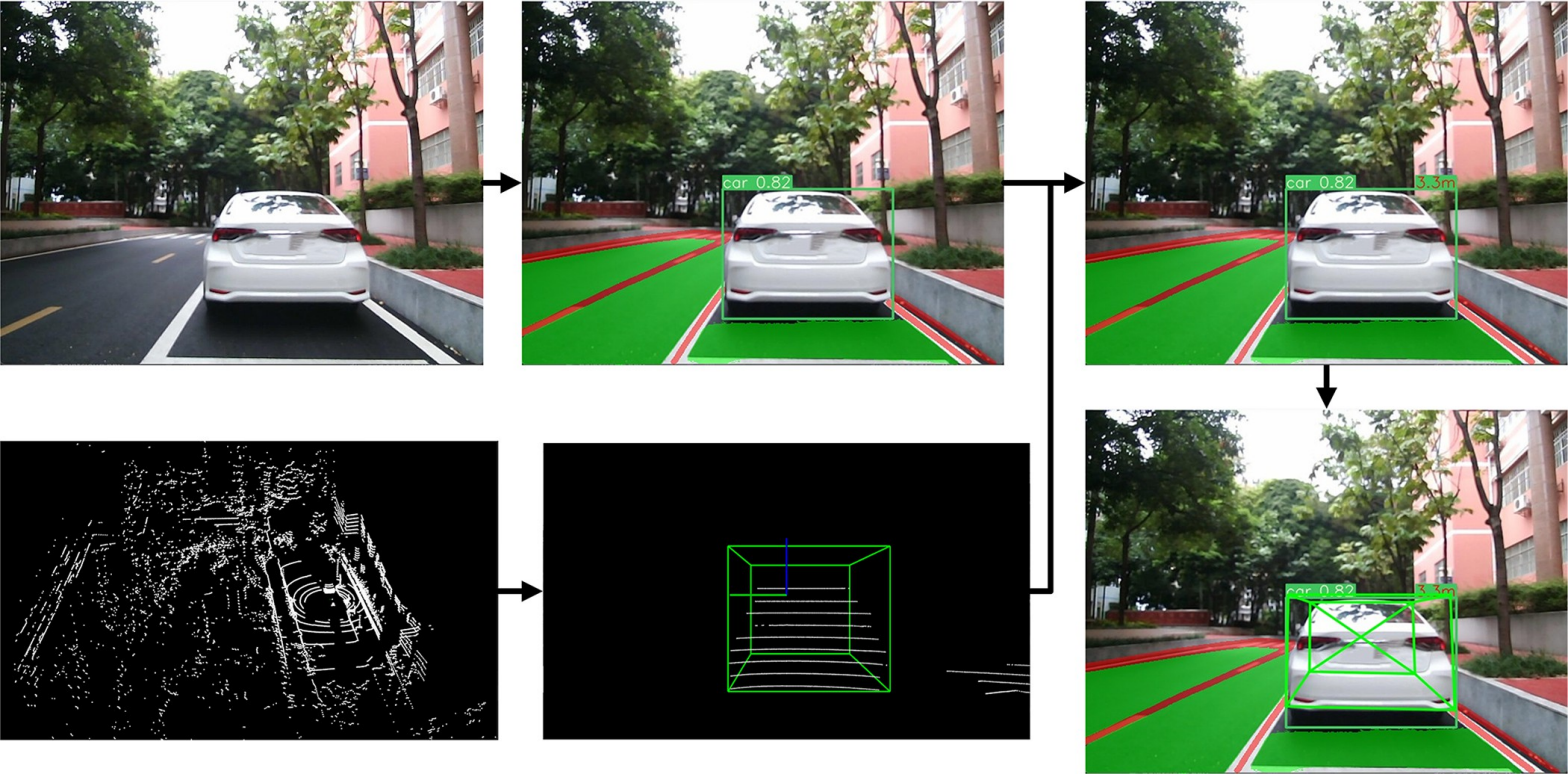
Fusion perception visualization results.

The fusion of perception visualization results is depicted in Fig 20. The top left of the predicted bounding boxes provides information regarding the class and confidence of the perceived object, while the top right displays distance information acquired from lidar perception. The class and confidence information of the predicted bounding boxes play a crucial role in identifying the types and potential levels of danger of surrounding objects, thereby influencing driving decisions and ensuring safety. Combining the 3D predicted bounding boxes obtained from lidar perception with the corresponding 2D predicted bounding boxes acquired from visual perception allows for improved accuracy in object recognition, tracking, pose estimation, and precise localization in traffic scenarios, thereby enhancing the perception capabilities and safety of the autonomous driving system. The distance information obtained from lidar perception measures the spatial relationship between perceived objects and the vehicle, facilitating obstacle avoidance and path planning.

**Fig 21:**
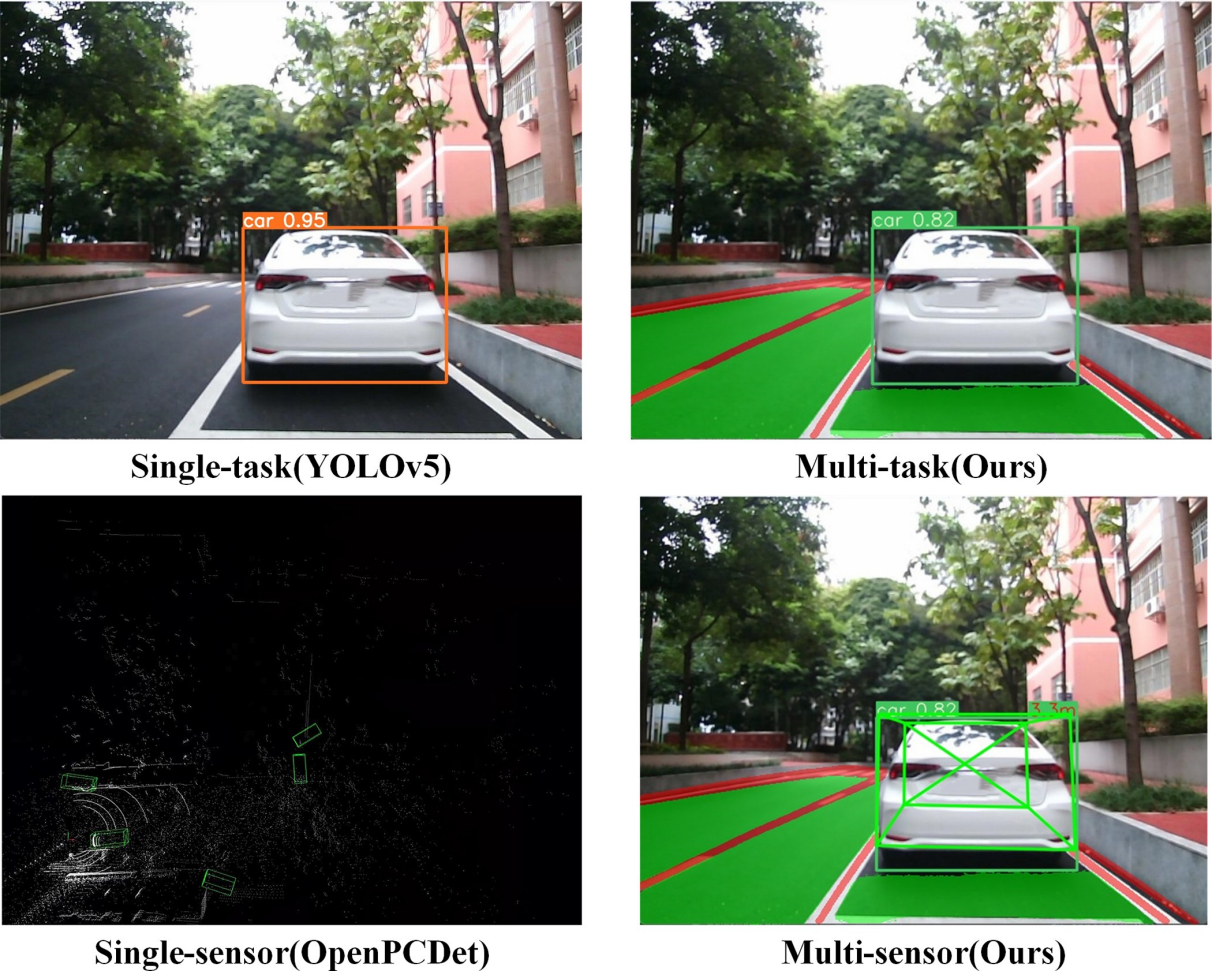
Comparative analysis of visualization results across various variants.

To emphasize the performance of our model, we compared the visualization results in various scenarios, including single-task and multi-task as well as single-sensor and multi-sensor setups, as depicted in Fig 21. In the single-task scenario, the YOLOv5 object detection algorithm was employed, while the OpenPCDet [48] lidar 3D object detection algorithm was used in the single-sensor scenario. Fig 21 illustrates that YOLOv5 solely detects the object category, whereas our multi-task model not only identifies object categories but also detects lane lines and drivable areas. Conversely, OpenPCDet only detects 3D objects and lacks the ability to precisely perceive relevant information such as object distance and category. Therefore, the proposed multi-task perception fusion algorithm, integrating lidar and visual sensors, enables vehicles to attain more comprehensive and accurate perception results, thus enhancing the panoramic driving system’s understanding of the surrounding environment.

## Conclusion

The presented research introduces a panoramic driving perception fusion algorithm hinged on multi-task learning. The experimental results demonstrate that this algorithm exhibits exceptional detection performance not only on the BDD100K dataset but also on the KITTI dataset, outperforming the majority of CNN-based and transformer-based models. Furthermore, it showcases improved overall performance, high accuracy, and robustness. The fusion technique, which synergizes lidar and visual sensors, significantly augments the holistic perception and comprehension of the ambient environment. Lidar’s proficiency in delivering pinpoint distance metrics is instrumental in sculpting precise environmental networks and obstacle detection. Concurrently, visual sensors excel in discerning objects, lane demarcations, and navigable terrains. The amalgamation of data and characteristics from both lidar and visual sensors markedly enhances perception accuracy and robustness, effectively addressing the challenge of achieving precise panoramic driving perception on limited hardware resources. This provides a fundamental basis of support for applications such as autonomous driving and intelligent connected vehicles.

Looking forward, endeavors will concentrate on refining the fusion algorithm’s structure, bolstering perception accuracy, and its eventual integration into real-world autonomous driving ecosystems. Additionally, avenues like the confluence of lidar, millimeter-wave radar, and visual sensor data, alongside multi-target trajectory tracking, will be explored. Such investigative trajectories aim to amplify the efficacy and applicability of panoramic driving perception, catalyzing advancements in autonomous driving innovations.
